# Infectious Shock and Toxic Shock Syndrome Diagnoses in Hospitals, Colorado, USA

**DOI:** 10.3201/eid1911.121547

**Published:** 2013-11

**Authors:** Michael A. Smit, Ann-Christine Nyquist, James K. Todd

**Affiliations:** Warren Alpert Medical School of Brown University, Providence, Rhode Island, USA (M.A. Smit);; University of Colorado School of Medicine, Aurora, Colorado, USA (A.-C. Nyquist, J.K. Todd);; Colorado School of Public Health, Aurora (A.-C. Nyquist, J.K. Todd);; Children’s Hospital Colorado, Aurora (A.-C. Nyquist, J.K. Todd)

**Keywords:** toxic shock syndrome, infectious shock, Staphylococcus aureus, group A streptococcus, epidemiology, surveillance, bacteria, Colorado

## Abstract

In Colorado, USA, diagnoses coded as toxic shock syndrome (TSS) constituted 27.3% of infectious shock cases during 1993–2006. The incidence of staphylococcal TSS did not change significantly overall or in female patients 10–49 years of age but increased for streptococcal TSS. TSS may be underrecognized among all ages and both sexes.

First described in 1978, toxic shock syndrome (TSS) is a severe febrile illness now confirmed to be caused by exotoxin-producing strains of *Staphyloccocus aureus* and *Streptococcus pyogenes* (*1*). Investigations based on extensive chart review and/or microbiology laboratory data suggest little or no decrease in overall TSS incidence and an increase in streptococcal TSS (*2*–*4*). Given the persistence and severity of TSS and the differences in its treatment from other causes of septic shock, its evolving epidemiology needs to be accurately monitored (*5*–*7*). To this end, we assessed International Classification of Diseases, Ninth revision, Clinical Modification (ICD-9-CM)–coded TSS cases in Colorado, USA.

## The Study

In 2007, we queried the Colorado Hospital Association database for ICD-9-CM codes that identified diagnoses consistent with infectious shock or TSS unrelated to pregnancy or childbirth (*8*). The study population comprised Colorado residents 1–65 years of age, selected by ZIP code of residence, who were discharged from Colorado hospitals during 1993–2006. Presumptive cases of “infectious shock” were 1) TSS or meningococcal shock of any diagnostic code (040.82, 040.89, 036.3); 2) principal diagnosis of hypotension or shock (785.50, 785.59, 998.0, 458.0, 796.3) plus any secondary diagnosis of staphyloccocal infection (038.1x, 041.1x, 482.4x); streptococcal infection (041.0x, 482.3x, 034.0, 038.1); scarlet fever (034.1); or bacteremia, septicemia, or other infection (code list available from authors); and 3) principal diagnosis of bacteremia, septicemia, staphyloccocal infection, streptococcal infection, other infection, or scarlet fever, plus any secondary diagnostic code of shock (see above codes). Infectious shock was further grouped into 3 code categories: 1) TSS-specific code: code for TSS (040.82 or 040.89) in any diagnostic field; 2) possible TSS code: infectious shock code without a specific code for TSS but with a code for infection with *S. aureus* (038.11, 041.11, 042.41) or *S. pyogenes*(041.01, 482.31) or with scarlet fever (034.1); and 3) infectious shock code, not TSS: infectious shock not otherwise classified. TSS was further designated as “strep” if it was associated with any code for *S. pyogenes*; all other TSS cases were assumed to be caused by *S. aureus* and were designated as “staph.” All case definitions were based on ICD-9-CM codes assigned by the discharging hospital.

Annual population-based incidences during 1993–2006 were calculated as cases per 100,000 persons by using extrapolated estimates of population by age interval and sex based on the US 1990 and 2000 censuses (*9*). Annual numbers of TSS cases reported to the State of Colorado were obtained from the Colorado Department of Public Health and Environment and classified as either TSS associated with *S. pyogenes* (reporting began in 2000) or TSS (assumed otherwise to be associated with *S. aureus* infection).

**Figure 1:**
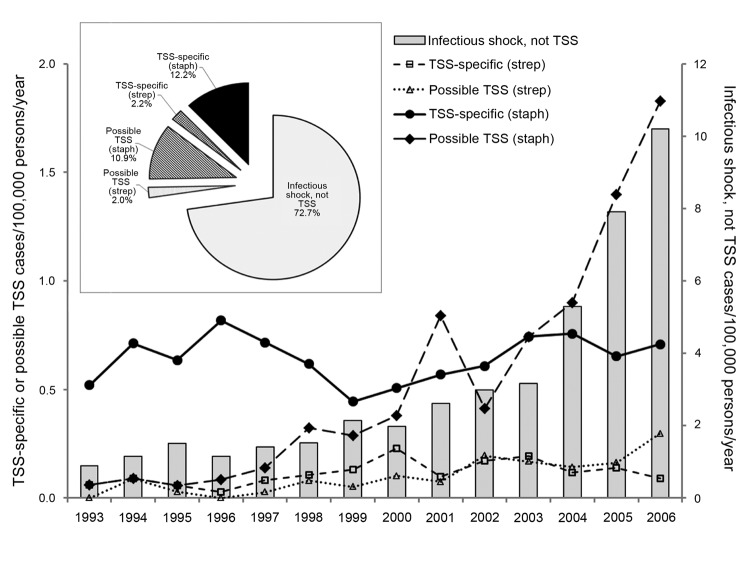
Yearly rates of International Classification of Diseases, Ninth Revision, Clinical Modification–coded infectious shock, Colorado, 1993–2006. Insert: cumulative proportion of cases. TSS, toxic shock syndrome; strep, streptococci; staph, staphylococci.

Infectious shock incidence increased significantly from 1993 through 2006 (R^2^ = 0.708, p<0.001 by linear regression). Of the 2,861 hospitalized persons with infectious shock, those assigned TSS-specific codes accounted for 411 (14.4%), and possible TSS codes constituted 371 (13.0%) (Figure 1). Of the 782 TSS-specific and possible TSS cases, 121 (15.5%) had a diagnostic code related to *S. pyogenes*; the remaining 661 (84.5%) cases were assumed by default to be associated with *S. aureus*. Case-fatality rates were significantly lower (p<0.001) for TSS-specific cases (5.6%) than for infectious shock, not TSS (29.3%). The incidence of TSS-specific (staph) cases did not change significantly from 1993 through 2006, whereas incidences for all other categories significantly increased. Beginning in 2000, an annual average of 54% (range 22%–100%) of TSS-specific (strep) and 16% (range 8%–26%) of TSS-specific (staph) cases were annually passively reported to the Colorado Department of Public Health and Environment.

**Figure 2:**
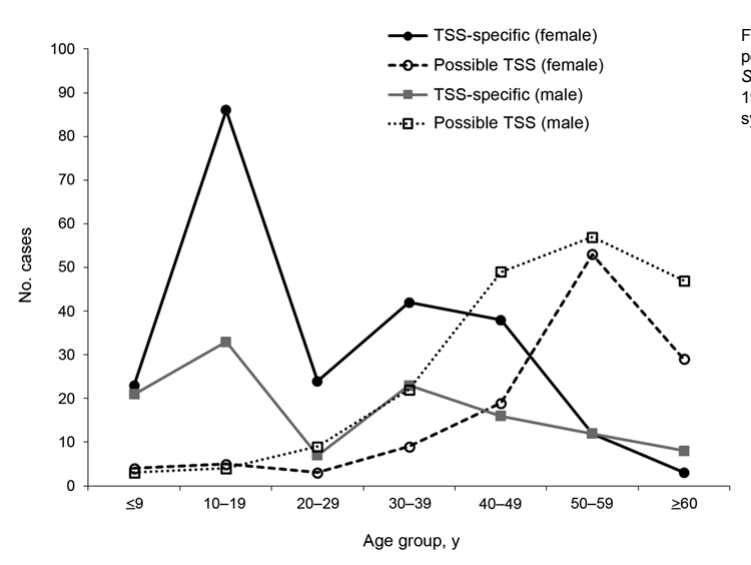
Total TSS-specific and possible TSS codes associated with *Staphylococcus aureus*, Colorado, 1993–2006. TSS, toxic shock syndrome.

The ages and sexes of patients assigned codes for TSS-specific and possible TSS (staph) is shown in Figure 2. Most cases occurred in female patients 10–49 years of age (peak 10 −19 years). For both sexes, the proportion of cases was comparable for the <10-year and >49-year age groups.

The crude population-based incidence for TSS-specific (staph) cases in patients 1–65 years of age averaged 0.64 per 100,000 (95% CI 0.59–0.70). For female patients 10–39 years of age, the incidence averaged 1.18 per 100,000 (95% CI 0.94–1.41). We compared the annual incidence for TSS-specific (staph) cases in Colorado during 1993–2006 (this study) with estimated incidences from 2 previously reported periods in Colorado using medical record review for 1970–1982 and 1987–1997 (*4*,*5*). We found no significant difference in the annual incidence in female patients 10–39 years of age among the 3 periods (p = 0.134, analysis of variance).

Fifty-three case records (1.8% of total sample) of patients hospitalized during 1998–2006 at Children’s Hospital Colorado were identified with TSS-specific, possible TSS, or infectious shock not TSS codes and available for independent, blinded review using the Centers for Disease Control and Prevention’s staphylococcal and streptococcal TSS case definitions (*10*). Within this subset, our ICD-9-CM–based code definition for TSS-specific or possible TSS had a sensitivity of 86.5%, specificity of 75.0%, and positive predictive value of 88.9%.

## Conclusions

Martin et al. reported that the incidence of septic shock increased from 1979 through 2000 in the United States (*11*). Using similar methods, we showed an increase in infectious shock in Colorado from 1993 through 2006 and estimated that the diagnosis of TSS accounted for as much as 27% of all cases of infectious shock. The TSS-specific incidence attributed to *S. aureus* has remained relatively constant in Colorado, although TSS attributed to *S. pyogenes* appears to be increasing. The latter has been noted in other studies as well (*12*,*13*). The observation that TSS accounts for a substantial proportion of all infectious shock is of clinical importance because TSS may respond to therapies (e.g., clindamycin, intravenous immune globulin, steroids) not ordinarily used for septic shock caused by other organisms, with more favorable outcomes as evidenced by significantly lower case-fatality rates for TSS noted in our study (*5*–*8*). For the current study period of 1993–2006, <20% of cases with a specific ICD-9-CM diagnosis of TSS (presumed staphylococcal) were reported to the state’s passive surveillance system. Passive surveillance systems may be of limited use if reported cases are not numerous enough to track trends (*14*). Our data show that the estimated incidence for staphylococcal TSS has not decreased significantly in Colorado since 1980 (*3*,*4*). Data recently published using similar methods also reported stable TSS incidence (*15*).

Our methods have important limitations. Our chart validation of coded definitions demonstrated reasonable sensitivity and positive predictive value for TSS-specific and possible TSS codes at a single institution; however, discharge coding among institutions is not necessarily consistent or precise and can result in ascertainment errors when applied to larger discharge populations. The TSS-specific code definition most likely underestimates the true incidence of less severe disease variants. Discharge databases contain little additional data that would facilitate risk factor assessment.

With these limitations, our results suggest that surveillance of TSS with a hospital discharge database provides significantly more sensitive case ascertainment than conventional passive reporting. Electronic definitions that use population-based databases could improve identification of TSS cases with a better understanding of its epidemiology. Given the clinical and management differences between shock caused by TSS, *S. aureus*, *S. pyogenes*, and other organisms, it is important to recognize TSS as a common cause of infectious shock in persons of both sexes and all ages.
